# Supplementary material for: Dengue, Zika, and Chikungunya viral circulation and hospitalization rates in Brazil from 2014 to 2019: An ecological study
Source: PLoS Negl Trop Dis. 2022 Jul 27;16(7):e0010602. doi: 10.1371/journal.pntd.0010602 (PMC9359537; doi:10.1371/journal.pntd.0010602)
Supplement: S1 Table — (DOCX) [file pntd.0010602.s001.docx]

**S1 Table.** Studied chapters and subcauses of hospitalizations.

|  | **Group** | **ICD-10 codes** |
| --- | --- | --- |
| **Arboviral related causes of hospitalization** | Dengue (all) | A90-A91 |
|  | Dengue (classic) | A90 |
|  | Dengue haemorragic | A91 |
|  | Arthropod-borne viral fevers and viral haemorrhagic fevers | A92-A99 |
|  | Chikungunya virus disease | A92.0 |
|  | Zika virus disease | A92.5 |
| **Major causes of hospitalizations (chapters)** | Chapter III: Diseases of the blood and blood-forming organs and certain disorders involving the immune mechanism | D50-D89 |
|  | Chapter IV: Endocrine, nutritional and metabolic diseases | E00-E89 |
|  | Chapter V: Mental and behavioural disorders | F01-F99 |
|  | Chapter VI: Diseases of the nervous system | G00-G09 |
|  | Chapter VII: Diseases of the eye and adnexa | H00-H59 |
|  | Chapter IX: Diseases of the circulatory system | I00-I99 |
|  | Chapter X: Diseases of the respiratory system | J00-J99 |
|  | Chapter XI: Diseases of the digestive system | K00-K95 |
|  | Chapter XII: Diseases of the skin and subcutaneous tissue | L00-L99 |
|  | Chapter XIII: Diseases of the musculoskeletal system and connective tissue | M00-M99 |
|  | Chapter XIV: Diseases of the genitourinary system | N00-N99 |
| **Cause specific hospitalizations** | Diabetes mellitus | E10-E13 |
|  | Cerebrovascular diseases | I60-I69 |
|  | Hypertensive diseases | I10-I15 |
|  | Postviral fatigue syndrome | G93.3 |
|  | Ischemic heart diseases | I20-I25 |
|  | Inflammatory diseases of the central nervous system | G00-G09 |
|  | Encephalitis, myelitis and encephalomyelitis; Encephalitis, myelitis and encephalomyelitis in diseases classified elsewhere | G04-G05 |
|  | Sequelae of inflammatory diseases of central nervous system | G09 |
|  | Inflammatory polyneuropathy (including [Guillain-Barré](https://www.medicinanet.com.br/cid10/5792/g610_sindrome_de_guillain_barre.htm)) | G61 |
|  | Acute myocarditis | I40 |
|  | Arthropathies | M00-M25 |
|  | Pregnancy with abortive outcome | O00-O08 |

^1^ https://icd.who.int/browse10/2019/en#/S00-S09
