# Supplementary material for: Dengue, Zika, and Chikungunya viral circulation and hospitalization rates in Brazil from 2014 to 2019: An ecological study
Source: PLoS Negl Trop Dis. 2022 Jul 27;16(7):e0010602. doi: 10.1371/journal.pntd.0010602 (PMC9359537; doi:10.1371/journal.pntd.0010602)
Supplement: S2 Table — (DOCX) [file pntd.0010602.s002.docx]

**S2 Table.** Mean yearly notifications per 100,000 inhabitants during the studied considering as the denominator all municipalities and considering only those who in a given month had at least one case of the disease.

|  | **Mean yearly arboviruses notifications/1,000** | | | | | |
| --- | --- | --- | --- | --- | --- | --- |
|  | **DENV** | | **ZIKV** | | **CHIKV** | |
|  | **All municipalities** | **Municipalities >=1 case** | **All municipalities** | **Municipalities >=1 case** | **All municipalities** | **Municipalities >=1 case** |
| ***North*** |  |  |  |  |  |  |
| Rondônia | 3.19 | 3.25 | 0.06 | 14.08 | 0.03 | 6.23 |
| Acre | 17.86 | 18.25 | 0.03 | 7.12 | 0.08 | 18.33 |
| Amazonas | 1.71 | 1.92 | 0.21 | 35.88 | 0.01 | 2.54 |
| Roraima | 5.18 | 5.18 | 0.12 | 23.74 | 1.23 | 158.43 |
| Pará | 1.18 | 1.28 | 0.07 | 21.45 | 0.36 | 65.06 |
| Amapá | 2.61 | 2.69 | 0.08 | 13.97 | 0.12 | 16.25 |
| Tocantins | 10.55 | 11.28 | 0.26 | 61.62 | 0.31 | 72.87 |
| ***Northeast*** |  |  |  |  |  |  |
| Maranhão | 1.00 | 1.27 | 0.11 | 35.34 | 0.32 | 66.46 |
| Piauí | 2.10 | 2.65 | 0.01 | 3.26 | 0.41 | 116.39 |
| Ceará | 6.45 | 6.50 | 0.06 | 14.42 | 2.62 | 370.31 |
| Rio Grande do Norte | 3.63 | 3.91 | 0.02 | 5.05 | 0.59 | 103.03 |
| Paraíba | 3.26 | 3.72 | 0.05 | 15.05 | 0.45 | 88.61 |
| Pernambuco | 5.68 | 5.86 | 0.01 | 1.98 | 0.32 | 49.44 |
| Alagoas | 4.39 | 4.50 | 0.23 | 43.47 | 0.58 | 103.16 |
| Sergipe | 2.40 | 2.63 | 0.01 | 1.96 | 0.36 | 67.27 |
| Bahia | 2.04 | 2.39 | 0.30 | 77.02 | 0.25 | 57.70 |
| ***Southeast*** |  |  |  |  |  |  |
| Minas Gerais | 11.92 | 12.84 | 0.08 | 26.04 | 0.22 | 57.79 |
| Espírito Santo | 9.10 | 9.14 | 0.14 | 26.20 | 0.13 | 22.24 |
| Rio de Janeiro | 2.07 | 2.08 | 0.55 | 70.25 | 1.13 | 151.23 |
| São Paulo | 9.68 | 9.78 | 0.02 | 4.81 | 0.01 | 1.42 |
| ***South*** |  |  |  |  |  |  |
| Paraná | 6.31 | 6.65 | 0.01 | 3.58 | 0.00 | 1.23 |
| Santa Catarina | 0.86 | 1.00 | 0.00 | 1.25 | 0.00 | 1.32 |
| Rio Grande do Sul | 0.26 | 0.33 | 0.00 | 1.57 | 0.00 | 0.86 |
| ***Central-west*** |  |  |  |  |  |  |
| Mato Grosso do Sul | 11.35 | 11.47 | 0.12 | 27.66 | 0.03 | 5.94 |
| Mato Grosso | 4.73 | 4.95 | 0.91 | 145.19 | 0.82 | 159.90 |
| Goiás | 15.71 | 15.85 | 0.24 | 52.69 | 0.00 | 1.01 |
| Distrito Federal | 5.11 | 5.12 | 0.02 | 1.95 | 0.01 | 1.33 |
