# Supplementary material for: Dengue, Zika, and Chikungunya viral circulation and hospitalization rates in Brazil from 2014 to 2019: An ecological study
Source: PLoS Negl Trop Dis. 2022 Jul 27;16(7):e0010602. doi: 10.1371/journal.pntd.0010602 (PMC9359537; doi:10.1371/journal.pntd.0010602)
Supplement: S3 Table — (DOCX) [file pntd.0010602.s003.docx]

**S3 Table.** Descriptive of monthly dengue, zika and chikungunya incidence and age-standardized hospitalization rates in the 5570 Brazilian municipalities from 2014 to 2019.

| **Study variables** | **Mean (SD)** | **Median [range]** |
| --- | --- | --- |
| ***Arboviral diseases notifications^1^*** |  |  |
| Dengue incidence (cases/100,000) | 41.00 (191.62) | 0.00 [0.00, 14191.22] |
| Zika incidence (cases/100,000) | 0.50 (15.16) | 0.00 [0.00, 3606.12] |
| Chykungunya incidence (cases/100,000) | 1.27 (24.27) | 0.00 [0.00, 2952.03] |
|  |  |  |
| ***Hospitalization rates*** |  |  |
| *Arboviral direct complications* |  |  |
| Arthropod-borne viral fevers and viral haemorrhagic fevers (A92-A99) | 0.15 (2.77) | 0.00 [0.00, 371.36] |
| Chikungunya virus disease (A92.0) | 0.04 (1.49) | 0.00 [0.00, 288.65] |
| Zika virus disease (A92.5)* | 0.00 (0.00) | 0.00 [0.00, 0.00] |
| Dengue (all) (A90-A91) | 2.64 (15.86) | 0.00 [0.00, 2234.09] |
| Dengue (classic) (A90) | 2.58 (15.76) | 0.00 [0.00, 2234.09] |
| Dengue haemorragic (A91) | 0.06 (0.98) | 0.00 [0.00, 183.03] |
|  |  |  |
| *Indirect complications* |  |  |
| All causes | 497.05 (236.79) | 467.90 [0.00, 5000.08] |
|  |  |  |
| *By causes* |  |  |
| Diabetes mellitus (E10-E13) | 3.23 (7.73) | 0.00 [0.00, 233.18] |
| Cerebrovascular diseases (I60-I69) | 7.52 (10.34) | 4.59 [0.00, 224.44] |
| Hypertensive diseases (I10-I15) | 5.00 (13.80) | 0.00 [0.00, 754.44] |
| Ischemic heart diseases (I20-I25) | 8.84 (13.14) | 4.62 [0.00, 581.38] |
| Inflammatory diseases of the central nervous system (G00-G09) | 0.32 (2.44) | 0.00 [0.00, 224.98] |
| Encephalitis, myelitis and encephalomyelitis; Encephalitis, myelitis and encephalomyelitis in diseases classified elsewhere (G04-G05) | 0.05 (0.80) | 0.00 [0.00, 113.03] |
| Sequelae of inflammatory diseases of central nervous system (G09) | 0.06 (1.16) | 0.00 [0.00, 151.98] |
| Acute myocarditis (I40) | 0.03 (0.78) | 0.00 [0.00, 122.62] |
| Arthropathies (M00-M25) | 0.00 (0.04) | 0.00 [0.00, 25.26] |
| Inflammatory polyneuropathy (including [Guillain-Barré](https://www.medicinanet.com.br/cid10/5792/g610_sindrome_de_guillain_barre.htm)) (G61) | 3.89 (10.67) | 0.00 [0.00, 487.20] |
| Pregnancy with abortive outcome | 0.19 (1.93) | 0.00 [0.00, 271.67] |
| Inflammatory diseases of the central nervous system (G00-G09) | 7.11 (10.73) | 2.64 [0.00, 216.45] |
|  |  |  |
| By chapters |  |  |
| Diseases of the blood and blood-forming organs and certain disorders involving the immune mechanism (D50-D89) | 4.38 (8.58) | 0.00 [0.00, 314.76] |
| Endocrine, nutritional and metabolic diseases (E00-E89) | 12.73 (19.12) | 6.65 [0.00, 732.12] |
| Diseases of the circulatory system (I00-I99) | 44.56 (36.72) | 37.83 [0.00, 1000.10] |
| Mental and behavioural disorders (F01-F99) | 14.42 (26.11) | 4.71 [0.00, 775.95] |
| Diseases of the nervous system (G00-G99) | 8.32 (29.93) | 2.49 [0.00, 3661.76] |
| Diseases of the eye and adnexa (H00-H59) | 2.79 (8.48) | 0.00 [0.00, 594.31] |
| Diseases of the respiratory system (J00-J99) | 60.07 (67.30) | 41.63 [0.00, 2576.45] |
| Diseases of the digestive system (K00-K95) | 49.00 (40.16) | 41.96 [0.00, 1347.44] |
| Diseases of the skin and subcutaneous tissue (L00-L99) | 8.78 (15.22) | 3.66 [0.00, 788.48] |
| Diseases of the musculoskeletal system and connective tissue (M00-M99) | 9.31 (16.62) | 3.98 [0.00, 605.32] |
| Diseases of the genitourinary system (N00-N99) | 37.05 (35.31) | 29.47 [0.00, 860.34] |

^1^ In municipalities that registered at least one case of arboviral disease during the study period.
