# Supplementary material for: Dengue, Zika, and Chikungunya viral circulation and hospitalization rates in Brazil from 2014 to 2019: An ecological study
Source: PLoS Negl Trop Dis. 2022 Jul 27;16(7):e0010602. doi: 10.1371/journal.pntd.0010602 (PMC9359537; doi:10.1371/journal.pntd.0010602)
Supplement: S4 Table — (DOCX) [file pntd.0010602.s004.docx]

**S4 Table.** Descriptive of monthly dengue, zika and chikungunya incidence and age-standardized hospitalization rates in the 27 Brazilian states.

| **Study variables** | **Mean (SD)** | **Median [range]** |
| --- | --- | --- |
| ***Arboviral diseases notifications^1^*** |  |  |
| Dengue incidence (cases/100,000) | 46.42 (88.24) | 16.92 [0.13, 1056.98] |
| Zika incidence (cases/100,000) | 1.15 (7.52) | 0.03 [0.00, 192.32] |
| Chykungunya incidence (cases/100,000) | 3.19 (17.48) | 0.10 [0.00, 409.71] |
|  |  |  |
| ***Hospitalization rates*** |  |  |
| *Arboviral direct complications* |  |  |
| Arthropod-borne viral fevers and viral haemorrhagic fevers (A92-A99) | 0.13 (0.28) | 0.04 [0.00, 4.16] |
| Chikungunya virus disease (A92.0) | 0.04 (0.15) | 0.00 [0.00, 2.21] |
| Zika virus disease (A92.5)* | 0.00 (0.00) | 0.00 [0.00, 0.00] |
| Dengue (all) (A90-A91) | 2.15 (3.10) | 1.04 [0.00, 29.47] |
| Dengue (classic) (A90) | 2.06 (2.96) | 0.98 [0.00, 28.00] |
| Dengue haemorragic (A91) | 0.09 (0.25) | 0.03 [0.00, 5.31] |
|  |  |  |
| *Indirect complications* |  |  |
| All causes | 470.05 (86.34) | 473.26 [1.44, 858.67] |
|  |  |  |
| *By causes* |  |  |
| Diabetes mellitus (E10-E13) | 2.96 (1.25) | 2.82 [0.00, 14.54] |
| Cerebrovascular diseases (I60-I69) | 0.35 (0.28) | 0.28 [0.00, 1.76] |
| Hypertensive diseases (I10-I15) | 0.07 (0.10) | 0.05 [0.00, 0.80] |
| Ischemic heart diseases (I20-I25) | 0.09 (0.23) | 0.00 [0.00, 1.14] |
| Inflammatory diseases of the central nervous system (G00-G09) | 0.04 (0.08) | 0.03 [0.00, 1.49] |
| Encephalitis, myelitis and encephalomyelitis; Encephalitis, myelitis and encephalomyelitis in diseases classified elsewhere (G04-G05) | 0.00 (0.00) | 0.00 [0.00, 0.02] |
| Sequelae of inflammatory diseases of central nervous system (G09) | 2.84 (1.58) | 2.47 [0.00, 9.18] |
| Acute myocarditis (I40) | 0.16 (0.12) | 0.15 [0.00, 1.31] |
| Arthropathies (M00-M25) | 9.54 (3.08) | 9.02 [0.02, 31.37] |
| Inflammatory polyneuropathy (including [Guillain-Barré](https://www.medicinanet.com.br/cid10/5792/g610_sindrome_de_guillain_barre.htm)) (G61) | 0.03 (0.21) | 0.00 [0.00, 4.26] |
| Pregnancy with abortive outcome | 47.61 (14.51) | 46.88 [0.22, 100.26] |
| Inflammatory diseases of the central nervous system (G00-G09) | 3.72 (1.26) | 3.71 [0.01, 9.37] |
|  |  |  |
| By chapters |  |  |
| Diseases of the blood and blood-forming organs and certain disorders involving the immune mechanism (D50-D89) | 12.31 (8.22) | 11.08 [0.00, 38.34] |
| Endocrine, nutritional and metabolic diseases (E00-E89) | 7.13 (3.00) | 6.65 [0.03, 16.97] |
| Diseases of the circulatory system (I00-I99) | 3.04 (2.34) | 2.48 [0.00, 15.95] |
| Mental and behavioural disorders (F01-F99) | 47.07 (16.56) | 45.03 [0.10, 103.55] |
| Diseases of the nervous system (G00-G99) | 42.72 (9.19) | 42.80 [0.13, 84.51] |
| Diseases of the eye and adnexa (H00-H59) | 10.29 (4.22) | 9.53 [0.02, 36.74] |
| Diseases of the respiratory system (J00-J99) | 7.43 (3.36) | 6.74 [0.02, 19.55] |
| Diseases of the digestive system (K00-K95) | 31.83 (8.34) | 31.85 [0.09, 60.92] |
| Diseases of the skin and subcutaneous tissue (L00-L99) | 7.52 (26.60) | 1.11 [0.00, 493.67] |
| Diseases of the musculoskeletal system and connective tissue (M00-M99) | 3.61 (16.25) | 0.19 [0.00, 303.81] |
| Diseases of the genitourinary system (N00-N99) | 55.96 (103.3) | 20.29 [0.14, 1075.68 |

^2^ In municipalities that registered at least one case of arboviral disease during the study period.
