# Supplementary material for: Dengue, Zika, and Chikungunya viral circulation and hospitalization rates in Brazil from 2014 to 2019: An ecological study
Source: PLoS Negl Trop Dis. 2022 Jul 27;16(7):e0010602. doi: 10.1371/journal.pntd.0010602 (PMC9359537; doi:10.1371/journal.pntd.0010602)
Supplement: S6 Table — (DOCX) [file pntd.0010602.s006.docx]

**S6 Table.** Changes of monthly age-standardized hospitalization rates associated with Zika in municipalities with at least 200 case of the disease considering an effect at the same month, and with 1 or 2 months delay.

| **Municipality level Zika incidence** | **Monthly basis** | **1 month lag** | **2 month lag** |
| --- | --- | --- | --- |
|  | **RR^1^ (95%** Crl**)** | **RR^1^ (95%** Crl**)** | **RR^1^ (95%** Crl**)** |
| **All causes** | 1.0001 (1-1.0002) | 0.9997 (0.9992-1.0002) | 0.9999 (0.9996-1.0002) |
| **By chapter** |  |  |  |
| Diseases of the blood and blood-forming organs and certain disorders involving the immune mechanism (D50-D89) | 1.0002 (1-1.0004) | 1.0008 (0.9997-1.002) | 0.9994 (0.9983-1.0005) |
| Endocrine, nutritional and metabolic diseases (E00-E89) | 1.0003 (1.0001-1.0004) | 1.0005 (0.9996-1.0015) | 0.999 (0.9983-0.9997) |
| Diseases of the circulatory system (I00-I99) | 1.0001 (1-1.0002) | 1.0006 (1-1.0012) | 1 (0.9997-1.0004) |
| Mental and behavioural disorders (F01-F99) | 1.0003 (1-1.0005) | 0.9966 (0.9943-0.999) | 0.9995 (0.9986-1.0005) |
| Diseases of the nervous system (G00-G99) | 1.0001 (0.9999-1.0003) | 0.9975 (0.996-0.999) | 0.9994 (0.9986-1.0001) |
| Diseases of the eye and adnexa (H00-H59) | 0.9995 (0.9988-1.0001) | 0.9964 (0.9915-1.0017) | 0.9981 (0.9957-1.0004) |
| Diseases of the respiratory system (J00-J99) | 1 (0.9999-1.0002) | 1.0007 (1-1.0014) | 0.9999 (0.9995-1.0004) |
| Diseases of the digestive system (K00-K95) | 1.0001 (1-1.0002) | 0.9996 (0.999-1.0002) | 1.0002 (0.9998-1.0006) |
| Diseases of the skin and subcutaneous tissue (L00-L99) | 1.0001 (0.9999-1.0003) | 0.9988 (0.9977-0.9998) | 1.0008 (1.0002-1.0014) |
| Diseases of the musculoskeletal system and connective tissue (M00-M99) | 1 (0.9998-1.0002) | 0.9994 (0.9982-1.0005) | 0.9998 (0.9991-1.0004) |
| Diseases of the genitourinary system (N00-N99) | 1.0001 (1-1.0003) | 0.9995 (0.9989-1.0001) | 0.9999 (0.9995-1.0004) |
| **By arboviruses diseases** |  |  |  |
| Dengue (A90-A91) | 1.0002 (0.9986-1.0019) | 1.0058 (0.9978-1.0151) | 1.0067 (0.9992-1.0157) |
| Dengue non-hemorragic (A90) | 1.0001 (0.9985-1.002) | 1.0056 (0.9975-1.0149) | 1.0083 (1.0001-1.0182) |
| Dengue haemorragic (A91) | 1.0032 (0.9949-1.0124) | 1.0138 (0.9875-1.044) | 1 (0-1.568525E+85) |
| Arthropod-borne viral fevers and viral haemorrhagic fevers (A92-A99) | 1.0076 (0.9441-1.082) | 1.0069 (0.9396-1.0755) | 1.0033 (0.9657-1.0465) |
| **By indirect causes** |  |  |  |
| Diabetes mellitus (E10-E13) | 1.0004 (1.0002-1.0006) | 1.001 (0.9996-1.0024) | 0.9962 (0.9939-0.9982) |
| Cerebrovascular diseases (I60-I69) | 1.0002 (1-1.0003) | 0.9999 (0.9989-1.0009) | 0.9996 (0.999-1.0003) |
| Hypertensive diseases (I10-I15) | 1.0002 (0.9999-1.0005) | 1.0003 (0.9982-1.0024) | 1.0006 (0.9996-1.0016) |
| Ischemic heart diseases (I20-I25) | 1 (0.9998-1.0002) | 1.0004 (0.9994-1.0013) | 1 (0.9994-1.0005) |
| Inflammatory diseases of the central nervous system (G00-G09) | 1.0024 (0.9994-1.0052) | 0.9992 (0.9931-1.0056) | 1.0016 (0.9945-1.0088) |
| Encephalitis, myelitis and encephalomyelitis; Encephalitis, myelitis and encephalomyelitis in diseases classified elsewhere (G04-G05) | 1.006 (0.9937-1.0181) | 0.9945 (0.979-1.0096) | 1.0026 (0.9909-1.0134) |
| Sequelae of inflammatory diseases of central nervous system (G09) | 1.0041 (0.9975-1.0096) | 0.9994 (0.9915-1.0082) | 1.0034 (0.9931-1.0128) |
| Acute myocarditis (I40) | 0.9995 (0.9796-1.0198) | 0.9997 (0.9717-1.0285) | 0.9993 (0.9688-1.0306) |
| Arthropathies (M00-M25) | 0.9999 (0.9995-1.0002) | 0.9978 (0.9955-1.0001) | 1.0002 (0.9992-1.0012) |
| Inflammatory polyneuropathy (including [Guillain-Barré](https://www.medicinanet.com.br/cid10/5792/g610_sindrome_de_guillain_barre.htm)) (G61) | 1.0082 (1.0033-1.0133) | 0.9995 (0.9898-1.0097) | 0.9908 (0.9778-1.0032) |
| Pregnancy with abortive outcome (O00-O08) | 1.0001 (0.9999-1.0003) | 0.9994 (0.9984-1.0004) | 0.9999 (0.9993-1.0006) |

**^1^** Adjusted for the Human Development Index, Gini Index and coverage of the family health Strategy.
