# Supplementary material for: Dengue, Zika, and Chikungunya viral circulation and hospitalization rates in Brazil from 2014 to 2019: An ecological study
Source: PLoS Negl Trop Dis. 2022 Jul 27;16(7):e0010602. doi: 10.1371/journal.pntd.0010602 (PMC9359537; doi:10.1371/journal.pntd.0010602)
Supplement: S7 Table — (DOCX) [file pntd.0010602.s007.docx]

**S7 Table.** Changes of monthly age-standardized hospitalization rates associated with chikungunya in municipalities with at least 200 case of the disease considering an effect at the same month, and with 1 or 2 months delay.

| **Municipality level Chikungunya incidence** | **Monthly basis** | **1 month lag** | **2 month lag** |
| --- | --- | --- | --- |
|  | **RR^1^ (95%** Crl**)** | **RR^1^ (95%** Crl**)** | **RR^1^ (95%** Crl**)** |
| **All causes** | 1 (1-1.0001) | 1 (0.9999-1.0001) | 1 (0.9999-1) |
| **By chapter** |  |  |  |
| Diseases of the blood and blood-forming organs and certain disorders involving the immune mechanism (D50-D89) | 1.0003 (1.0001-1.0004) | 1.0002 (1.0001-1.0004) | 0.9998 (0.9995-1.0001) |
| Endocrine, nutritional and metabolic diseases (E00-E89) | 1 (0.9999-1.0001) | 1.0001 (0.9999-1.0003) | 0.9998 (0.9995-1) |
| Diseases of the circulatory system (I00-I99) | 1 (1-1.0001) | 0.9999 (0.9998-1) | 0.9999 (0.9998-1) |
| Mental and behavioural disorders (F01-F99) | 0.9999 (0.9997-1) | 0.9998 (0.9996-1.0001) | 0.9997 (0.9994-0.9999) |
| Diseases of the nervous system (G00-G99) | 1 (0.9999-1.0001) | 0.9999 (0.9997-1.0001) | 1 (0.9998-1.0002) |
| Diseases of the eye and adnexa (H00-H59) | 0.9998 (0.9994-1.0003) | 0.9996 (0.999-1.0003) | 0.9998 (0.9991-1.0005) |
| Diseases of the respiratory system (J00-J99) | 1 (1-1.0001) | 1 (0.9999-1.0001) | 1 (0.9998-1.0001) |
| Diseases of the digestive system (K00-K95) | 1 (0.9999-1) | 0.9999 (0.9997-1) | 0.9998 (0.9997-0.9999) |
| Diseases of the skin and subcutaneous tissue (L00-L99) | 1 (0.9999-1.0001) | 1.0002 (1-1.0003) | 1 (0.9998-1.0001) |
| Diseases of the musculoskeletal system and connective tissue (M00-M99) | 0.9999 (0.9997-1) | 1 (0.9999-1.0002) | 1.0001 (0.9999-1.0003) |
| Diseases of the genitourinary system (N00-N99) | 0.9999 (0.9999-1) | 1 (0.9999-1.0001) | 1 (0.9998-1.0001) |
| **By arboviruses diseases** |  |  |  |
| Dengue (A90-A91) | 1.0044 (1.0037-1.0051) | 0.9999 (0.9992-1.0007) | 1.0002 (0.9992-1.0013) |
| Dengue non-hemorragic (A90) | 1.0044 (1.0037-1.0051) | 0.9999 (0.9992-1.0006) | 1.0004 (0.9993-1.0015) |
| Dengue haemorragic (A91) | 1.0028 (1.0004-1.0057) | 1.0063 (0.9968-1.0181) | 0.9608 (0.9307-0.9874) |
| Arthropod-borne viral fevers and viral haemorrhagic fevers (A92-A99) | 1.0067 (1.0034-1.0111) | 0.9963 (0.9876-1.0067) | 1.016 (1-1.0361) |
| **By indirect causes** |  |  |  |
| Diabetes mellitus (E10-E13) | 1.0001 (0.9999-1.0002) | 1.0002 (0.9999-1.0005) | 0.9999 (0.9995-1.0002) |
| Cerebrovascular diseases (I60-I69) | 1 (0.9999-1.0001) | 1 (0.9998-1.0002) | 1.0001 (0.9999-1.0003) |
| Hypertensive diseases (I10-I15) | 0.9999 (0.9997-1.0001) | 0.9998 (0.9995-1.0002) | 1 (0.9994-1.0005) |
| Ischemic heart diseases (I20-I25) | 1 (0.9998-1.0001) | 0.9999 (0.9997-1.0001) | 0.9998 (0.9996-1) |
| Inflammatory diseases of the central nervous system (G00-G09) | 1.0002 (0.9995-1.001) | 0.9998 (0.998-1.0014) | 0.998 (0.9959-0.9998) |
| Encephalitis, myelitis and encephalomyelitis; Encephalitis, myelitis and encephalomyelitis in diseases classified elsewhere (G04-G05) | 0.9995 (0.9984-1.0007) | 1.0021 (0.9979-1.0067) | 0.9962 (0.992-0.9999) |
| Sequelae of inflammatory diseases of central nervous system (G09) | 1.0057 (0.998-1.0135) | 0.9976 (0.9915-1.0035) | 1.0058 (0.9966-1.0154) |
| Acute myocarditis (I40) | 1.0068 (0.9927-1.0204) | 0.996 (0.9771-1.015) | 1.0032 (0.9801-1.0263) |
| Arthropathies (M00-M25) | 0.9998 (0.9996-1.0001) | 1 (0.9997-1.0002) | 1.0003 (1-1.0006) |
| Inflammatory polyneuropathy (including [Guillain-Barré](https://www.medicinanet.com.br/cid10/5792/g610_sindrome_de_guillain_barre.htm)) (G61) | 1.0003 (0.9989-1.0017) | 1 (0.9971-1.0026) | 0.9889 (0.9813-0.9956) |
| Pregnancy with abortive outcome (O00-O08) | 0.9999 (0.9998-1) | 0.9999 (0.9997-1) | 1 (0.9998-1.0001) |

**^1^** Adjusted for the Human Development Index, Gini Index and coverage of the family health Strategy.
