# Supplementary material for: Dengue, Zika, and Chikungunya viral circulation and hospitalization rates in Brazil from 2014 to 2019: An ecological study
Source: PLoS Negl Trop Dis. 2022 Jul 27;16(7):e0010602. doi: 10.1371/journal.pntd.0010602 (PMC9359537; doi:10.1371/journal.pntd.0010602)
Supplement: S9 Table — (DOCX) [file pntd.0010602.s009.docx]

**S9 Table.** Changes of monthly age-standardized hospitalization rates associated with Zika in the 27 Brazilian States considering an effect at the same month, and with 1 or 2 months delay.

| **State level Zika incidence** | **Monthly basis** | **1 month lag** | **2 month lag** |
| --- | --- | --- | --- |
|  | **RR (95%CI)** | **RR (95%CI)** | **RR (95%CI)** |
| **All causes** | 1 (0.9987-1.0015) | 1.0005 (0.9991-1.0019) | 0.9992 (0.9978-1.0006) |
| **By chapter** |  |  |  |
| Diseases of the blood and blood-forming organs and certain disorders involving the immune mechanism (D50-D89) | 1.0003 (0.9988-1.0019) | 1.0008 (0.9993-1.0023) | 1.0006 (0.9991-1.0022) |
| Endocrine, nutritional and metabolic diseases (E00-E89) | 1.0011 (0.9997-1.0026) | 1.0005 (0.9991-1.002) | 0.9991 (0.9976-1.0005) |
| Diseases of the circulatory system (I00-I99) | 0.9999 (0.9985-1.0014) | 1.0004 (0.999-1.0019) | 0.9988 (0.9974-1.0003) |
| Mental and behavioural disorders (F01-F99) | 1.0007 (0.9987-1.0028) | 1.0006 (0.9987-1.0026) | 0.9962 (0.9943-0.9982) |
| Diseases of the nervous system (G00-G99) | 0.9993 (0.9977-1.0009) | 1.001 (0.9994-1.0026) | 0.9988 (0.9972-1.0004) |
| Diseases of the eye and adnexa (H00-H59) | 1.0005 (0.9978-1.0034) | 1.0006 (0.9979-1.0035) | 0.999 (0.9963-1.002) |
| Diseases of the respiratory system (J00-J99) | 1.0007 (0.999-1.0025) | 1.0009 (0.9993-1.0027) | 0.9989 (0.9972-1.0007) |
| Diseases of the digestive system (K00-K95) | 1.0001 (0.9987-1.0016) | 1.0007 (0.9992-1.0022) | 0.9994 (0.998-1.001) |
| Diseases of the skin and subcutaneous tissue (L00-L99) | 1.0009 (0.9993-1.0025) | 1.0011 (0.9995-1.0027) | 0.999 (0.9974-1.0006) |
| Diseases of the musculoskeletal system and connective tissue (M00-M99) | 1.0001 (0.9984-1.0019) | 1.0002 (0.9986-1.002) | 0.9986 (0.9969-1.0003) |
| Diseases of the genitourinary system (N00-N99) | 1 (0.9985-1.0014) | 1.0006 (0.9992-1.0021) | 0.9986 (0.9972-1.0001) |
| **By arboviruses diseases** |  |  |  |
| Dengue (A90-A91) | 1.0052 (0.9994-1.0118) | 0.997 (0.9922-1.0025) | 1.0028 (0.9978-1.0086) |
| Dengue non-hemorragic (A90) | 1.0052 (0.9994-1.0118) | 0.9969 (0.9921-1.0024) | 1.0027 (0.9977-1.0085) |
| Dengue haemorragic (A91) | 1.0032 (0.9944-1.0132) | 0.9996 (0.9927-1.0077) | 1.0159 (1.0016-1.0315) |
| Arthropod-borne viral fevers and viral haemorrhagic fevers (A92-A99) | 1.003 (0.996-1.0111) | 0.9932 (0.9853-1.0014) | 0.9993 (0.9918-1.0072) |
| Chikungunya virus disease (A92.5) | 1.0237 (0.9929-1.0589) | 1.0528 (0.9897-1.1324) | 1.0454 (0.9896-1.1077) |
| **By indirect causes** |  |  |  |
| Diabetes mellitus (E10-E13) | 1.0014 (0.9997-1.0031) | 0.9991 (0.9976-1.0007) | 0.999 (0.9973-1.0007) |
| Cerebrovascular diseases (I60-I69) | 1.0001 (0.9986-1.0018) | 1.0002 (0.9987-1.0018) | 0.9994 (0.9978-1.001) |
| Hypertensive diseases (I10-I15) | 0.9993 (0.9976-1.001) | 1.0008 (0.9992-1.0025) | 0.9987 (0.9971-1.0005) |
| Ischemic heart diseases (I20-I25) | 0.9991 (0.9976-1.0007) | 1.0004 (0.9988-1.002) | 0.9986 (0.997-1.0002) |
| Inflammatory diseases of the central nervous system (G00-G09) | 0.9991 (0.9962-1.0021) | 1.0011 (0.9988-1.0035) | 1.0012 (0.9984-1.004) |
| Encephalitis, myelitis and encephalomyelitis; Encephalitis, myelitis and encephalomyelitis in diseases classified elsewhere (G04-G05) | 0.9995 (0.9921-1.0068) | 1.0016 (0.9972-1.006) | 1.0024 (0.9985-1.0063) |
| Sequelae of inflammatory diseases of central nervous system (G09) | 1.0027 (0.9935-1.0125) | 0.9988 (0.9946-1.0035) | 0.9998 (0.9806-1.0173) |
| Acute myocarditis (I40) | 1.002 (0.9956-1.0078) | 1.0014 (0.9966-1.0061) | 0.9946 (0.9871-1.0014) |
| Arthropathies (M00-M25) | 0.9999 (0.998-1.0019) | 0.9998 (0.9979-1.0018) | 0.9977 (0.9958-0.9996) |
| Inflammatory polyneuropathy (including [Guillain-Barré](https://www.medicinanet.com.br/cid10/5792/g610_sindrome_de_guillain_barre.htm)) (G61) | 1.0054 (1.0025-1.0084) | 0.9991 (0.9962-1.002) | 1 (0.9963-1.0035) |
| Pregnancy with abortive outcome (O00-O08) | 0.9997 (0.9982-1.0012) | 1.0005 (0.999-1.0021) | 1.0001 (0.9986-1.0017) |
