# Supplementary material for: Dengue, Zika, and Chikungunya viral circulation and hospitalization rates in Brazil from 2014 to 2019: An ecological study
Source: PLoS Negl Trop Dis. 2022 Jul 27;16(7):e0010602. doi: 10.1371/journal.pntd.0010602 (PMC9359537; doi:10.1371/journal.pntd.0010602)
Supplement: S10 Table — (DOCX) [file pntd.0010602.s010.docx]

**S10 Table.** Changes of monthly age-standardized hospitalization rates associated with chikungunya in the 27 Brazilian States considering an effect at the same month, and with 1 or 2 months delay.

| **State level Chikungunya incidence** | **Monthly basis** | **1 month lag** | **2 month lag** |
| --- | --- | --- | --- |
|  | **RR (95%CI)** | **RR (95%CI)** | **RR (95%CI)** |
| **All causes** | 1.0003 (0.9998-1.0009) | 1 (0.9994-1.0005) | 1 (0.9995-1.0006) |
| **By chapter** |  |  |  |
| Diseases of the blood and blood-forming organs and certain disorders involving the immune mechanism (D50-D89) | 1.0004 (0.9998-1.0011) | 0.9996 (0.999-1.0003) | 0.9995 (0.9989-1.0002) |
| Endocrine, nutritional and metabolic diseases (E00-E89) | 1.0001 (0.9995-1.0007) | 1 (0.9994-1.0006) | 1.0003 (0.9997-1.0009) |
| Diseases of the circulatory system (I00-I99) | 1.0003 (0.9998-1.0009) | 0.9995 (0.999-1.0001) | 0.9997 (0.9992-1.0003) |
| Mental and behavioural disorders (F01-F99) | 1.0005 (0.9996-1.0014) | 0.9996 (0.9989-1.0004) | 1.0003 (0.9995-1.0012) |
| Diseases of the nervous system (G00-G99) | 1 (0.9994-1.0007) | 0.9999 (0.9993-1.0005) | 1 (0.9993-1.0007) |
| Diseases of the eye and adnexa (H00-H59) | 1.0005 (0.999-1.002) | 0.9994 (0.9982-1.0007) | 1.0005 (0.9992-1.0019) |
| Diseases of the respiratory system (J00-J99) | 1.0005 (0.9999-1.0012) | 0.9999 (0.9993-1.0005) | 1.0002 (0.9995-1.0008) |
| Diseases of the digestive system (K00-K95) | 1.0003 (0.9997-1.0009) | 0.9998 (0.9993-1.0004) | 0.9998 (0.9992-1.0003) |
| Diseases of the skin and subcutaneous tissue (L00-L99) | 1.0008 (1.0001-1.0014) | 1.0002 (0.9996-1.0008) | 0.9998 (0.9992-1.0005) |
| Diseases of the musculoskeletal system and connective tissue (M00-M99) | 1.0001 (0.9994-1.0008) | 0.9999 (0.9993-1.0006) | 1.0001 (0.9994-1.0008) |
| Diseases of the genitourinary system (N00-N99) | 1.0003 (0.9997-1.0009) | 0.9998 (0.9993-1.0004) | 0.9998 (0.9992-1.0004) |
| **By arboviruses diseases** |  |  |  |
| Dengue (A90-A91) | 1.0061 (1.0035-1.009) | 1.0041 (1.0014-1.0071) | 0.998 (0.9957-1.0005) |
| Dengue non-hemorragic (A90) | 1.0064 (1.0037-1.0093) | 1.0043 (1.0016-1.0072) | 0.9981 (0.9958-1.0006) |
| Dengue haemorragic (A91) | 1.0022 (0.9995-1.0052) | 1.0014 (0.998-1.0051) | 0.995 (0.99-0.9996) |
| Arthropod-borne viral fevers and viral haemorrhagic fevers (A92-A99) | 1.0283 (1.0212-1.0359) | 1.0053 (1.0007-1.0104) | 0.9982 (0.9952-1.0016) |
| Chikungunya virus disease (A92.5) | 1.0206 (1.0115-1.031) | 1.014 (1.0038-1.0258) | 0.9953 (0.9907-1.0001) |
| **By indirect causes** |  |  |  |
| Diabetes mellitus (E10-E13) | 1.0007 (1-1.0014) | 1.0001 (0.9994-1.0008) | 1.0003 (0.9996-1.001) |
| Cerebrovascular diseases (I60-I69) | 1.0005 (0.9999-1.0012) | 0.9996 (0.999-1.0003) | 0.9994 (0.9988-1.0001) |
| Hypertensive diseases (I10-I15) | 1.0001 (0.9994-1.0008) | 0.9992 (0.9984-0.9999) | 0.9994 (0.9987-1.0001) |
| Ischemic heart diseases (I20-I25) | 0.9999 (0.9993-1.0005) | 0.9995 (0.9989-1.0001) | 0.9995 (0.9989-1.0002) |
| Inflammatory diseases of the central nervous system (G00-G09) | 0.9998 (0.9987-1.0009) | 0.9994 (0.9982-1.0006) | 0.9985 (0.9971-0.9999) |
| Encephalitis, myelitis and encephalomyelitis; Encephalitis, myelitis and encephalomyelitis in diseases classified elsewhere (G04-G05) | 0.9991 (0.9976-1.0007) | 0.9999 (0.9978-1.0018) | 0.9994 (0.9967-1.002) |
| Sequelae of inflammatory diseases of central nervous system (G09) | 0.9961 (0.9892-1.0033) | 1.0036 (0.9995-1.008) | 0.988 (0.9795-0.9965) |
| Acute myocarditis (I40) | 1 (0.9963-1.0032) | 0.9976 (0.9936-1.0013) | 0.9996 (0.9973-1.0019) |
| Arthropathies (M00-M25) | 0.9996 (0.9988-1.0004) | 0.9998 (0.9991-1.0006) | 0.9999 (0.9991-1.0008) |
| Inflammatory polyneuropathy (including [Guillain-Barré](https://www.medicinanet.com.br/cid10/5792/g610_sindrome_de_guillain_barre.htm)) (G61) | 1.0006 (0.9993-1.002) | 0.9989 (0.9975-1.0004) | 1.0023 (1.0007-1.0039) |
| Pregnancy with abortive outcome (O00-O08) | 0.9996 (0.999-1.0002) | 0.9996 (0.999-1.0002) | 0.9998 (0.9993-1.0004) |
